# Assessment of Safety and Prophylactic Efficacy of the EpiVacCorona Peptide Vaccine for COVID-19 Prevention (Phase III)

**DOI:** 10.3390/vaccines11050998

**Published:** 2023-05-18

**Authors:** Alexander B. Ryzhikov, Evgeny A. Ryzhikov, Marina P. Bogryantseva, Svetlana V. Usova, Elena A. Nechaeva, Elena D. Danilenko, Stepan A. Pyankov, Andrey S. Gudymo, Anastasiya A. Moiseeva, Galina S. Onkhonova, Oleg V. Pyankov, Ekaterina S. Sleptsova, Nikita V. Lomakin, Veronika S. Vasilyeva, Mikhail V. Tulikov, Vitaly G. Gusarov, Andrey A. Pulin, Maria A. Balalaeva, Svetlana B. Erofeeva, Stanislav A. Terpigorev, Olga A. Rychkova, Ivan M. Petrov, Viktoriia Y. Delian, Vladimir V. Rafalskiy, Sergey V. Tyranovets, Elena V. Gavrilova, Rinat A. Maksyutov

**Affiliations:** 1 State Research Center of Virology and Biotechnology “VECTOR”, Rospotrebnadzor, 630559 Koltsovo, Russia; ryzhik@vector.nsc.ru (A.B.R.);; 2 Limited Liability Company “EpiVac“, 630559 Koltsovo, Russia; 3 Central Clinical Hospital, Administrative Directorate of the President of the Russian Federation, 121359 Moscow, Russia; 4 Pirogov National Medical and Surgical Center, 105203 Moscow, Russia; 5 Russian Research Center of Surgery n.a. Academician B.V. Petrovsky, 119435 Moscow, Russia; 6 Krasnogorsk City Hospital No. 1, 143403 Krasnogorsk, Russia; 7 Moscow Regional Research and Clinical Institute n.a. M.F. Vladimirsky, 129110 Moscow, Russia; 8 Institute of Motherhood and Childhood, Tyumen State Medical University, Ministry of Health of the Russian Federation, 625023 Tyumen, Russia; 9 Municipal Clinical Hospital No. 7, 420103 Kazan, Russia; 10 Medical Institute, Immanuel Kant Baltic Federal University, 236041 Kaliningrad, Russia; 11 Limited Liability Company “Clinical Research Laboratory”, 111672 Moscow, Russia

**Keywords:** SARS-CoV-2, COVID-19, peptide vaccine

## Abstract

The State Research Center of Virology and Biotechnology “VECTOR” of the Federal Service for the Oversight of Consumer Protection and Welfare (Rospotrebnadzor) has developed the peptide-based EpiVacCorona vaccine, which is the first synthetic peptide-based antiviral vaccine for mass immunization in international vaccinology. An early clinical trial (Phase I–II) demonstrated that the EpiVacCorona vaccine is a safe product. The “Multicenter double-blind, placebo-controlled, comparative, randomized trial to assess the tolerability, safety, immunogenicity and prophylactic efficacy of the EpiVacCorona COVID-19 vaccine based on peptide antigens in 3000 volunteers aged 18 years and older” was performed regarding vaccine safety. The key objectives of the study were to evaluate the safety and prophylactic efficacy of the two-dose EpiVacCorona vaccine administered via the intramuscular route. The results of the clinical study (Phase III) demonstrated the safety of the EpiVacCorona vaccine. Vaccine administration was accompanied by mild local reactions in ≤27% of cases and mild systemic reactions in ≤14% of cases. The prophylactic efficacy of the EpiVacCorona COVID-19 vaccine after the completion of the vaccination series was 82.5% (CI95 = 75.3–87.6%). The high safety and efficacy of the vaccine give grounds for recommending this vaccine for regular seasonal prevention of COVID-19 as a safe and effective medicinal product.

## 1. Introduction

Coronaviruses (Coronaviridae) belong to a large family of RNA-containing viruses capable of infecting both animals and humans [1,2,3]. In humans, coronaviruses can cause a number of diseases ranging from mild acute respiratory infection to severe acute respiratory syndrome. Like other respiratory viral infections, the causative agent of COVID-19, SARS-CoV-2, is mainly transmitted by respiratory droplets, through aerosols, contaminated objects, or by direct contact [4,5,6,7]. Coronaviruses have a broad tropism and, in addition to the respiratory tract, can affect the liver, the kidneys, the intestine, the nervous system, the heart, and the eyes [8,9,10,11,12,13]. Being capable of inducing apoptosis, coronaviruses cause necrosis of the affected tissues, and fibrotic lung scars remain after patients’ recovery. Coronaviruses exhibit a strong effect on cell membrane permeability, thus causing electrolyte imbalance and disrupting protein transport [10].

Over the past two decades, coronaviruses have been responsible for the epidemic outbreaks of two respiratory diseases: the Middle East respiratory syndrome and severe acute respiratory syndrome [14,15,16,17]. The emergence of the novel coronavirus proves that diseases caused by this virus family pose a threat to global health because of the pandemic and must be carefully monitored. Mass vaccination is the most reliable method for stopping the pandemic.

The active ingredient of the EpiVacCorona vaccine developed at the State Research Center of Virology and Biotechnology of the Federal Service for the Oversight of Consumer Protection and Welfare (Rospotrebnadzor) is a combination of synthetic peptides mimicking the protective sites of the SARS-CoV-2 S-protein that are conjugated to the carrier protein and adsorbed onto an aluminum-containing adjuvant (aluminum hydroxide) [18,19,20].

The results of the clinical trial (Phase I–II) demonstrate that the EpiVacCorona vaccine is a safe medicinal product. Vaccine administration is accompanied only by mild local reactions. No systemic reactions (body temperature elevation and allergic reactions) have been observed. The vaccine stimulates the production of specific anti-coronavirus antibodies after intramuscular administration of two doses with a 21-day interval in 100% of vaccinated subjects [21].

The “Multicenter double-blind, placebo-controlled, comparative, randomized trial to assess the tolerability, safety, immunogenicity and prophylactic efficacy of the EpiVacCorona COVID-19 vaccine based on peptide antigens in 3000 volunteers aged 18 years and older” was carried out for obtaining extended data on vaccine safety and efficacy, as well as the most frequent adverse drug reactions. The key objectives of the study were to evaluate the safety and prophylactic efficacy of the two-dose EpiVacCorona vaccine administered via the intramuscular route.

The unique formulation of this vaccine makes the study data from this work of potentially great interest to the larger community. Inactivated, vectored, mRNA-, and DNA-based vaccine products for COVID-19 have been described in the literature. These data add one more layer of additional vaccine platform strategy to vaccines designed to address COVID-19.

## 2. Materials and Methods

### 2.1. Study Design

The study was conducted in eight federal state budget-funded institutions of the Russian Federation. Local laboratories of research centers were the study sites.

The sample size required to evaluate vaccine effectiveness depends on a number of factors, such as expected vaccine effectiveness, desired precision in assessing vaccine effectiveness, and infection rates in the unvaccinated population [22]. The optimal design for assessing the effectiveness of vaccination involves randomization of an equal number of subjects in the groups of the candidate vaccine and the control group (1:1). In trials that use a control group not vaccinated against a disease, it may be appropriate to use unbalanced randomization (e.g., 2:1 or 3:1) so that the majority of subjects receive the vaccine candidate [23]. Alternative approved antiSARS-CoV-2 vaccine formulation for comparison was not available at the start of phase III clinical trials of the EpiVacCorona vaccine; therefore, in our trials, we reduced the number of volunteers in the control group by a factor of three compared to the vaccine candidate group (3:1), which was approved by the ethics committee. The predictive effectiveness of the vaccine was assumed at 70%. For vaccine effectiveness estimation, we assumed a power of 80% for 95 percent confidence intervals (α = 0.05). The attack rate was estimated in accordance with the weekly data on COVID-19 cases in the Russian Federation obtained from the Johns Hopkins University Coronavirus Resource Center «https://coronavirus.jhu.edu/map.html (accessed on 23 November 2020)» Based on these assumptions, the sample size for calculating the vaccine effectiveness for cohort studies was estimated at 1640 people, with 407 in unvaccinated control group and 1233 in vaccinated group [24].

**Figure 1:**
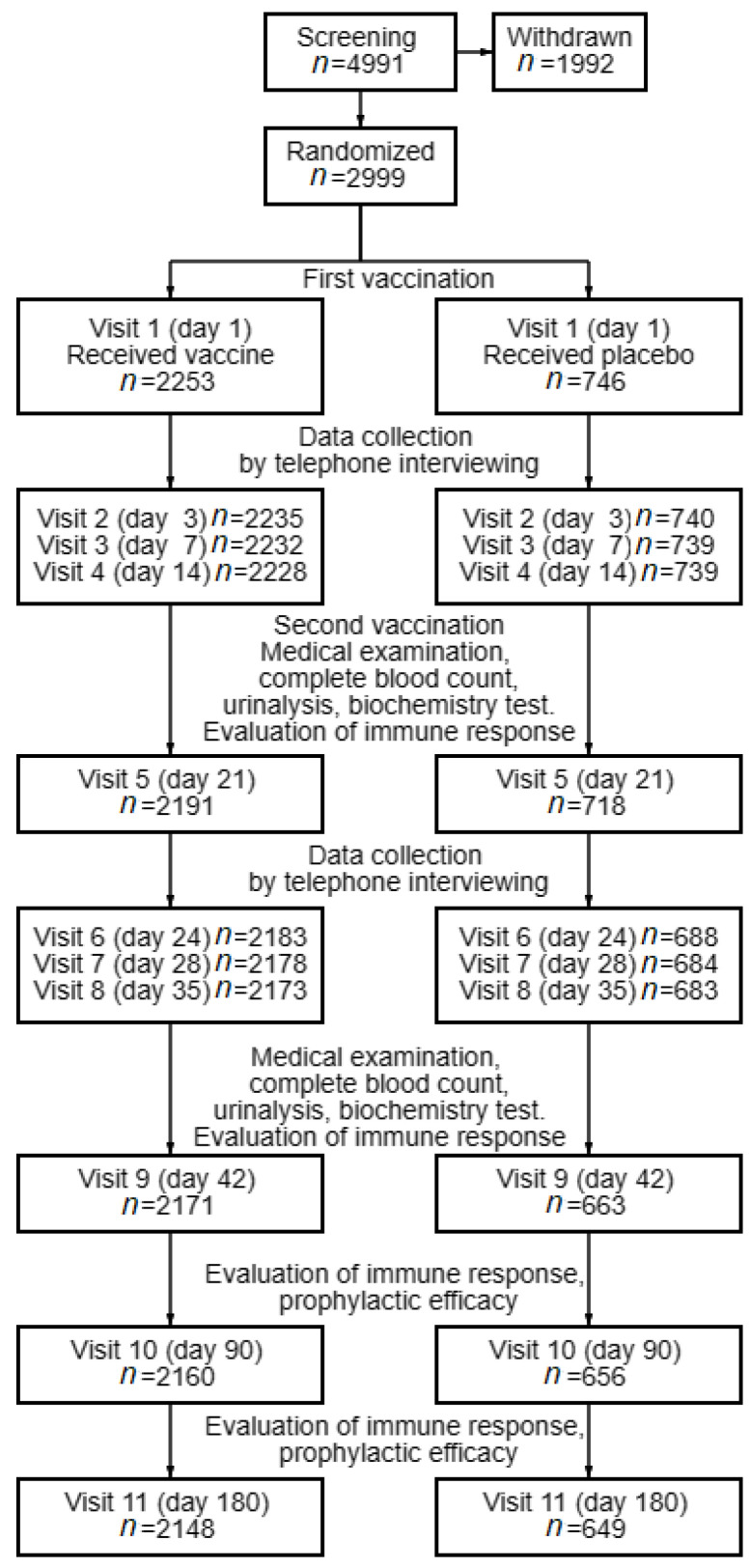
Schematic diagram of the study design.

A total of 4991 male and female volunteers aged 18 years or older were screened. Of those, 2999 volunteers met all the inclusion criteria, and none of the exclusion criteria were randomized (Figure 1); this far exceeds the calculated cohort size. After signing informed consent forms, the 2999 volunteers were allocated into groups at a 3:1 ratio. Group 1 consisted of 2253 subjects vaccinated intramuscularly with two doses of EpiVacCorona (0.5 mL each dose), and Group 2 involved 746 subjects vaccinated intramuscularly with two doses of placebo (0.5 mL each dose).

At each study site, the volunteers were randomly assigned to two groups. During screening, 1992 volunteers were withdrawn from the study because of non-compliance with the inclusion criteria or meeting the exclusion criteria.

Volunteers who had provided consent for participating in the clinical trial and met the inclusion criteria (stable vital signs; body mass index ranging from 18.5 to 30 kg/m^2^; attending all the scheduled visits, procedures, and examinations; and the use of effective methods of contraception throughout the entire study) were enrolled in the study.

The exclusion criteria were as follows: past history of the coronavirus infection; contacts with confirmed or suspected cases of SARS-CoV-2 infection one month prior to screening; positive PCR test result or positive test for anti-SARS-CoV-2 IgM or IgG detected at screening; current clinically and laboratory-confirmed diagnosis of SARS-CoV-2 or past history of such diagnosis; serious immunization reaction or complication of previous immunization; positive history of allergies; hypersensitivity to any drug ingredient; allergy to vaccine ingredients; vaccination with any vaccine within one month before randomization; vaccination with the rabies vaccines less than two months before randomization or scheduled vaccination with the rabies vaccines within one month after immunization using the study vaccines; pregnancy and breastfeeding; any acute respiratory disease less than three months before randomization; acute infectious or non-infectious diseases; exacerbation of chronic diseases less than four weeks before randomization; past history of pulmonary and extra pulmonary tuberculosis, cancer; autoimmune diseases, or skin conditions; long-term administration (>14 days) of immunosuppressants, systemic glucocorticoids or immunomodulatory drugs within six months before randomization; administration of immunoglobulin or blood-derived products within three months before randomization; severe comorbidities (decompensated diseases of internal organs or mental health disorders); and participation in other clinical trials during the period less than three months before randomization.

The withdrawal criteria included exacerbation of chronic liver, kidney, pancreatic, and heart diseases; occurrence of other infectious diseases (acute tonsillitis, hepatitis, etc.), cancer, or any other condition that may affect the study conduct or the subject’s condition in the Investigator’s opinion; patient’s pregnancy; voluntary withdrawal from the study or failure to adhere to the requirements for study participation.

This clinical study was a multicenter, double-blind, placebo-controlled, comparative randomized trial. The volunteers were allocated to study groups using envelopes. Before study initiation, sealed opaque envelopes within the Investigator Site File were provided to each study site in sufficient quantity. A randomization envelope contained information about the volunteer’s randomization ID and the name of the medicinal product to be administered. The investigational medicinal drugs were delivered to the study site in a ready-to-use (code-protected) form. To ensure blinding, before being injected, the investigational medicinal products were prepared for use behind a screen in the medical treatment room. Intramuscular injections of the medicinal products were delivered exclusively in the medical treatment room by qualified personnel (a vaccinator). After the first vaccination, all the volunteers were followed up by the Investigator for six months. Visits to the study sites were made on study day 1 (the first vaccination), day 21 (the second vaccination), day 42, day 90, and day 180. After the vaccination, volunteers stayed at the study site for 2 h under medical supervision. During the intervals between the first and second vaccination (Visit 1 and Visit 2), from day 2 until day 20, and during 21 days after the second vaccination, the Investigator collected information about any immunization reactions and concomitant treatment by telephone interviewing.

During the course of clinical trials, the number of active volunteers decreased due to the fact that compliance with the exclusion factors from the study was revealed or the volunteers voluntarily refused further participation, or the requirements for participation in the study were not met in the study.

An analysis of vaccine effectiveness was carried out for the per-protocol population. Vaccine safety was analyzed for the population of patients who had received at least one dose of the investigational medicinal product (the vaccine or placebo).

Prerequisites for conducting the clinical trial were as follows: Authorization No. 644 of the Ministry of Health of the Russian Federation dated 18 November 2020 (Addendum No. 4155830-20-1/D8 dated 18 November 2020 about the inclusion of additional study sites, Addendum No. 4156631-20-1/D8 dated 26 November 2020 about increasing the study sample size, and Addendum No. 4163382-20-1/PP about the new version of the Protocol and patient information leaflet) and Approval of the study by the Ethics Committee of the Russian Federation (Extract from the Protocol No. 256 dated 17 November 2020 and No. 265 dated 2 February 2021).

This clinical study was carried out in compliance with the principles outlined in the World Medical Association’s (WMA) Declaration of Helsinki (adopted by the 18th WMA Assembly, Helsinki, June 1964, in its most recent edition adopted by the 64th WMA Assembly, Fortaleza, October 2013), tripartite agreement on Good Clinical Practice (ICH GCP), as well as legislation of the Russian Federation controlling the conduct of clinical trials.

All the procedures (physical examination, clinical tests, immunologic tests, and detection of adverse events) were carried out in compliance with Protocol No. COV/pept-03/20 (Version 2.0 dated 20 January 2021). The study was initiated on 27 November 2020 and completed on 31 August 2021.

### 2.2. Vaccine and Placebo

The EpiVacCorona vaccine based on peptide antigens for COVID-19 prevention is a suspension for intramuscular injection, which has been designed and manufactured by the State Research Center of Virology and Biotechnology “VECTOR” of the Federal Service for the Oversight of Consumer Protection and Welfare (Rospotrebnadzor) (Koltsovo, Russia) (GMP-0112- 000170/17). Two doses of the vaccine were injected intramuscularly into volunteers with a 21–28-day interval. A single vaccine dose contained (225 ± 45) µg/0.5 mL active substance. The vaccine was injected into the deltoid muscle using sterile syringes. 

The vaccine was designed using synthetic peptide vaccine technology. It involves such stages as designing protective peptide fragments of the spike (S) protein, performing chemical synthesis of peptides, and conjugating the synthetic peptides with a carrier protein [18,19,20,21]. An original algorithm was used to design peptides potentially capable of eliciting a protective immune response against SARS-CoV-2. This investigation included analyzing the spatial structure of coronavirus S protein, reviewing and summarizing the data on linear and conformational epitopes as well as their properties, studying the amino acid variability of coronavirus S protein, identifying potential surface-exposed regions in the S-protein structure, and analyzing the amino acid composition of the peptides [18,19,20,21,25,26,27,28].

Synthetic peptides are covalently bound (conjugated) to a carrier protein. A recombinant chimeric carrier protein containing the SARS-CoV-2 nucleocapsid (N) protein was designed for the EpiVacCorona vaccine. The SARS-CoV-2 nucleocapsid protein was chosen because it is highly conserved and contains virus-specific T-cell epitopes [18,19,20]. The carrier protein covalently bound to synthetic peptides forms nanoparticles with a diameter of 30 nm. Aluminum hydroxide is a vaccine ingredient acting as an adjuvant.

Aluminum hydroxide in phosphate-buffered saline was used as a reference product (placebo). All the placebo ingredients were identical to those found in the vaccine except for the active ingredient, the peptide–protein complex, which is not contained in the placebo. The placebo was administered as two 0.5 mL intramuscular doses at a 21-day interval. The vaccine and placebo were stored in the temperature range of 2–8 °C.

### 2.3. Physical Examination and Clinical Laboratory Procedures

Physical examination, immunological, biochemistry, hematological analyses, and urinalysis were carried out according to standard clinical protocols. Vital signs (body temperature, blood pressure, heart rate, and respiratory rate) were assessed. The examination of volunteers also involved visual inspection of skin and mucous membranes (eyes, mouth, and the pharynx), auscultation, percussion of lungs, and palpation of the abdomen and lymph nodes.

The vital signs (blood pressure, heart rate, and respiratory rate) were monitored using conventional clinical methods. Blood pressure and heart rate were measured using an automated blood pressure monitor; the respiratory rate (breaths per minute) was measured either using a pneumotachometer or visually.

The immunization reactions were documented according to the data reported daily by the volunteer in the self-monitoring diary. The reactogenicity was assessed according to the number of systemic (elevated body temperature, malaise, headache, fatigue, excessive sweating, cough, sleep and appetite problems, nausea, vomiting, muscle and joint pain, bowel disorders, etc.) and local reactions (soreness at the injection site, hyperemia, swelling, pruritus, lymph node enlargement (for the cervical, axillary, and other lymph nodes), infiltrates, etc.). The volunteers documented all the immunization reactions daily in the self-monitoring diary.

Complete blood count, biochemistry test, and urinalysis were performed using unified methods. Complete blood count parameters (RBD count, WBC count, hemoglobin, platelet count, sedimentation rate, and WBC differential) were analyzed using automated hematology analyzers. Biochemistry parameters were studied on automated biochemistry analyzers using the photometry principle. Concentrations of proteins, trace elements, enzymes, and hormones in body fluids were measured photometrically using special reagent kits.

### 2.4. Safety Assessment

Vaccine administration causes a body’s response, which sometimes may have clinical manifestation. The typical immunization reactions can be either local or systemic. Local symptoms include hyperemia, swelling, soreness, infiltrate formation, and pruritus at the injection site. Systemic reactions manifest clinically as malaise, fever, and headache. 

The safety data were assessed according to frequency of adverse events. Adverse events were subdivided into immediate adverse events (allergic reactions) that occurred within 2 h after the vaccination and were detected both by the Investigator and according to information reported by the volunteer; adverse events (local and systemic reactions) that occurred within 7 days after the vaccination and were detected both by the Investigator and according to information reported by the volunteer; and adverse events that occurred 7 days after each vaccination (between day 8 and day 21 after each vaccination) and were detected both by the Investigator and according to information reported by the volunteer.

The frequency of serious adverse events was monitored throughout the entire follow-up period. A serious adverse event was defined as any untoward medical occurrence that: resulted in death; was life-threatening; required inpatient hospitalization of the volunteer or prolongation of existing hospitalization; resulted in persistent or significant disability/incapacity; or required intervention to prevent the outcomes listed above.

### 2.5. Detecting Coronavirus RNA in Nasopharyngeal Swabs

Detection of coronavirus RNA in nasopharyngeal swabs was performed using a Vector-PCRrv-2019-nCoV–RG kit for PCR detection of 2019-nCoV coronavirus RNA by fluorescence acquisition during hybridization phase according to the Technical Specifications TU 21.20.23-088-05664012-2020 (Market Authorization for the medical product No. RHS 2020/9677 dated 11 February 2020) manufactured at the State Research Center of Virology Biology “VECTOR” of the Federal Service for the Oversight of Consumer Protection and Welfare (Rospotrebnadzor) or a similar kit in accordance with the manufacturer’s instructions.

### 2.6. ELISA Kits for Quantifying Serum Antibodies

Specific antibodies against coronavirus antigens in human serum were quantified using a test kit for ELISA quantification of immunoglobulins in accordance with the manufacturer’s instructions. The level of IgM against SARS-CoV-2 antigens was analyzed using the SARS-CoV-2-IgM-ELISA-Best test kit, Marketing Authorization No. RHS 2020/10389. Manufacturer JSC “Vector-best”, Novosibirsk, Russia; the level of IgA against SARS-CoV-2 antigens was analyzed using the SARS-CoV-2-IgA-ELISA-Best test kit, Marketing Authorization No. RHS 2021/13633. Manufacturer JSC “Vector-best”, Novosibirsk, Russia; the level of IgG against antigens of inactivated SARS-CoV-2 virus was analyzed using the SARS-CoV-2-ELISA-Vector test kit according to the Technical Specifications TU 21.20.23-090-23548172-2020, Marketing Authorization No. RHS 2020/10017. Manufacturer State Research Center of Virology and Biotechnology “VECTOR” of the Federal Service for the Oversight of Consumer Protection and Welfare (Rospotrebnadzor), Novosibirsk, Russia; the level of IgG against SARS-CoV-2 antigens, the components of the EpiVacCorona vaccine, was analyzed using the SARS-CoV-2-IgG-Vector test kit according to the Technical Specifications TU 21.20.23-093-23548172-2020, Marketing Authorization No. RHS 2020/12952. Manufacturer State Research Center of Virology and Biotechnology “VECTOR” of the Federal Service for the Oversight of Consumer Protection and Welfare (Rospotrebnadzor), Novosibirsk, Russia. 

In order to normalize the results from different tests, a cut-off index (COI) was calculated, in which COI = (serum sample OD450)/(cut-off value).

### 2.7. Quantification of Virus-Neutralizing Antibodies in the Neutralization Assay

The presence of virus-neutralizing antibodies in volunteers’ serum was detected in the microneutralization assay by inhibition of focus-forming units in the monolayer culture of Vero E6 cells infected with Wuhan-like SARS-CoV-2 coronavirus variant, strain nCoV/Victoria/1/2020. The microneutralization assay of focus-forming units (ffu) is based on inhibition of the viral infection by antibodies in individual monolayer cells. The cells infected with the SARS-CoV-2 virus are visualized by immunochemical staining using antibodies specific to the virus protein (usually to the nucleoprotein NP). The number of stained cells (ffu) was counted under a microscope or using imaging systems. The neutralizing antibody titer was defined as the maximum dilution of the analyzed serum at which 50% of ffu in the well is inhibited compared to the control. The assay was carried out in accordance with the method described in [29] with some modifications.

### 2.8. Statistical Analysis

The demographic data and primary efficacy variables were assessed using parametric and nonparametric statistics. The main laboratory parameters evaluated during the study were analyzed using descriptive statistics. The values of these parameters were presented as the mean ± standard deviation or the median and interquartile range depending on the type of distribution of the parameter being assessed.

The safety data were evaluated according to the frequency of adverse events. The frequencies of adverse events and other traits in the groups (frequency analysis) were compared mostly using the χ^2^ test or Fisher’s exact test (if the expected trait frequency in one of the subgroups was <5). The severities of adverse events (mild, moderate, or severe) in the groups were compared using the nonparametric Mann–Whitney U test. The alpha values ≤0.05 were considered statistically significant. The required group size was calculated using the methods described in [30,31].

## 3. Results

### 3.1. Information about the Study Subjects

A total of 4991 volunteers were enrolled in the clinical study; of those, 1992 volunteers were withdrawn at screening because they failed to meet the inclusion criteria or met the exclusion criteria. A total of 2999 volunteers were randomized; of those, 2253 volunteers received the vaccine (Group 1), and 746 volunteers received a placebo (Group 2). 

Group 1 consisted of 52.7% males and 47.3% females. The male-to-female ratio in Group 2 was 49.6% and 50.4%, respectively. The mean age in Groups 1 and 2 was 49 ± 0.3 years and 46.5 ± 0.5 years, respectively. The mean body weight was 80.2 ± 0.44 kg in Group 1 and 78.8 ± 0.7 kg in Group 2. The mean height in the study groups was approximately the same (171.0 ± 0.19 cm in each group). The mean body mass index in both groups was 26.0 kg/m^2^.

A comparative analysis of the demographic and anthropometric parameters conducted using the Mann–Whitney U test revealed no significant intergroup differences in such parameters as sex, height, and BMI between volunteers who had received a placebo or the vaccine.

### 3.2. Safety and Reactogenicity of the EpiVacCorona Vaccine

The EpiVacCorona vaccine has previously passed phase I–II clinical trials in volunteers. During the entire follow-up period in phase I–II clinical trials, significant changes in body temperature, blood pressure, heart rate, or respiratory rate were detected in none of the volunteers who had received the EpiVacCorona vaccine or placebo. All the vital signs lay within the physiological range during all the periods. The vaccine caused no immediate adverse events (allergic reactions) within 2 h after the vaccination. No serious adverse events were observed during the study. The results of phase I–II clinical trials inferred that the EpiVacCorona vaccine was characterized by low reactogenicity, high safety profile, and good tolerability [21].

Phase III clinical trials of the EpiVacCorona vaccine were conducted to evaluate its safety and efficacy in 2999 volunteers. Intramuscular injection of the vaccine was accompanied by local and systemic body reactions. The local reactions manifested themselves as such mild symptoms as hyperemia, swelling, and soreness at the injection site. The systemic reactions manifested themselves as such mild symptoms as malaise, elevated body temperature, headache, and fatigue (Table 1).

Allergic reactions developing within 2 h after vaccination and local and systemic reactions developing during the period up to day seven after each vaccination were taken into account when assessing the reactogenicity.

Within 2 h after the first vaccination, two (0.09%) cases of allergic reactions were detected in the group of volunteers who had received the vaccine, and one (0.13%) case was detected in the placebo group. The local and systemic reactions within two hours were revealed in 44 (1.95%) volunteers who had received the vaccine and 16 (2.1%) volunteers who had received the placebo. Body temperature elevation within 2 h was observed in 15 (0.67%) vaccinated volunteers and 12 (1.6%) placebo recipients.

Within two hours after the second vaccination, one (0.05%) volunteer developed Quincke’s edema in the vaccinated group (the allergic reaction was stopped by prednisolone injection), and one (0.14%) volunteer in the placebo group had an allergic reaction. Within two hours after the second vaccination, local and systemic reactions were detected in 29 (1.4%) vaccinated volunteers and 9 (1.3%) placebo recipients. Elevated body temperature was detected in 13 (0.6%) volunteers who had received the vaccine and 7 (1.0%) placebo recipients.

Some volunteers reported that local reactions developed within the first two days after vaccination and lasted 2–4 days. These reactions typically develop and are differentiated within the first 3–4 days after vaccine administration. The results of statistical analysis revealed no significant intergroup differences in the analyzed parameters for the volunteers receiving a placebo and the vaccine. On day three after the first vaccination, soreness at the injection site was reported by 500 (22.4%) volunteers vaccinated with the EpiVacCorona vaccine and 183 (24.7%) placebo recipients. Injection site redness was observed in 58 (2.6%) volunteers vaccinated with the EpiVacCorona vaccine and 16 (2.2%) placebo recipients. Swelling at the injection site was observed in 50 (2.2%) and 17 (2.3%) cases in Group 1 and Group 2, respectively. The duration of local reactions ranged from 2 to 7 days. No statistically significant intergroup differences in analyzed parameters were revealed.

**Figure 2:**
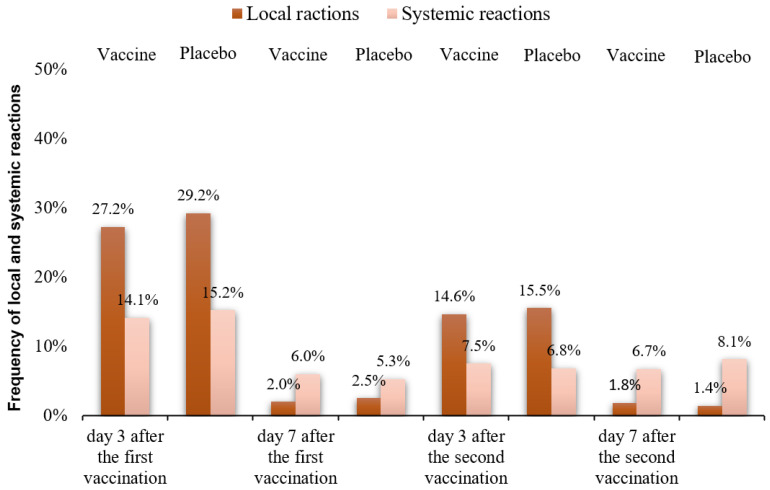
Frequency of local and systemic reactions in the groups of vaccinated volunteers and placebo recipients at different time after vaccination.

The second vaccination also triggered local reactions presenting as soreness, hyperemia, and swelling at the injection site. Thus, 271 (12.4%) volunteers were vaccinated with EpiVacCorona, and 89 (12.9%) placebo recipients complained of soreness at the injection site on day three after the vaccination. Hyperemia was reported by 22 (1.0%) volunteers in Group 1 and 9 (1.3%) volunteers in Group 2. Swelling was observed in 26 (1.2%) volunteers in Group 1 and 9 (1.3%) volunteers in Group 2. The local reactions were actually expected since the vaccine contains aluminum hydroxide. The World Health Organization’s (WHO’s) Global Advisory Committee on Vaccine Safety (GACVS) has proved that the local inflammatory reaction is associated with the long-term presence of aluminum at the injection site [32]. The local reactions were classified as mild and were not accompanied by worsening of the volunteer’s wellbeing. The severity of systemic reactions was assessed with allowance for acceptable reactions (elevated body temperature, fatigue, and headache). No statistically significant intergroup differences in the analyzed parameters were revealed during the entire follow-up period. Figure 2 summarizes the data on local and systemic reactions observed in the study groups.

Safety assessment of the vaccine also included evaluation of the patterns of changes in complete blood count, biochemistry, and urinalysis parameters, as well as vital signs.

Monitoring the vital signs during the entire follow-up period indicates that there were no intergroup differences in such parameters as the heart rate, diastolic blood pressure, and respiratory rate in volunteers vaccinated with the EpiVacCorona vaccine and placebo recipients. Volunteer monitoring during the study showed no changes in the functional status of the cardiovascular (arrhythmia, cardiodynia, or tachycardia), respiratory, gastrointestinal (nausea, stomach ache, or vomiting), and urogenital systems (impaired diuresis).

A comparative analysis of hematological parameters (RBC count, hemoglobin level, platelet count, WBC count; neutrophil count, eosinophil count, basophil count, monocyte count, lymphocyte count, and ESR) using the Mann–Whitney U test for independent samples revealed a statistically significant intergroup difference in several parameters for Groups 1 and 2. An increased basophil count was observed in Group 1 compared to Group 2 before vaccination, which still persisted on days 21, 42, and 180 after the first vaccination (*p* = 0.002, *p* = 0.02, *p* < 0.0001, and *p* = 0.006, respectively). The values of the parameter lay within the physiological range. On day 42 after the first vaccination, the eosinophil count in the group of vaccinated volunteers was increased compared with the placebo group (*p* < 0.0001); this increase lay within the physiological range. On day 42 after the first vaccination, an increase in the ESR was observed in Group 1 compared to Group 2 (*p* = 0.014). The parameter values lay within the physiological range. Elevated basophil count, urea, and CRP levels were observed in Group 1 before vaccination and are not associated with vaccination. However, elevated eosinophil count, ESR, and glucose levels detected on days 21 or 42 after the first vaccination can be associated with vaccination, and particularly, with allergic response.

Injection of vaccine products is accompanied by characteristic functional homeostatic shift. Therefore, the following biochemical parameters were analyzed to identify the potential adverse effects of the vaccine on the body’s functional status: alanine transaminase (ALT), aspartate transaminase (AST), lactate dehydrogenase, alkaline phosphatase (ALP), B-lipoproteins, cholesterol, total protein, total bilirubin, glucose, creatinine, urea, thymol turbidity test, and C-reactive protein (CRP).

The statistical analysis using the Mann–Whitney U test showed that the urea levels were increased in vaccinated volunteers compared to the placebo recipients before the vaccination, which persisted on days 21, 42, and 90 after the first vaccination (*p* = 0.003, *p* < 0.0001, *p* < 0.0001, and *p* < 0.0001, respectively). Meanwhile, these values lay within the physiological range. Statistical analysis revealed an increase in glucose levels in the group of vaccinated volunteers on days 21 and 42 after the first vaccination (*p* = 0.001 and *p* = 0.023, respectively). The mean values of the parameter lay within the physiological range. The CRP level was increased in the group of vaccinated volunteers before vaccination, persisting on days 21, 42, 90, and 180 after the first vaccination (*p* < 0.0001, *p* < 0.0001, *p* = 0.002, *p* = 0.001, and *p* = 0.040, respectively. The values of the parameter lay within the physiological range. Significant intergroup differences in alkaline phosphatase activity and creatinine and albumin levels were revealed at some visits. Meanwhile, the values of these parameters lay within the physiological range. The mean ALT and AST activities lay within the physiological range in both groups.

Adverse events, including serious ones, were reported during the clinical trials. Most of the general adverse events were acute respiratory viral infections (ARVIs) in both groups. Each case of serious adverse event was investigated to determine its causality, compliance with the clinical trial protocol, and compliance with the inclusion/exclusion criteria. Six cases of adverse events were excluded from the vaccine safety and efficacy assessment because of protocol violations or failure to comply with the inclusion, exclusion, or withdrawal criteria. A total of 632 adverse events were documented during the study; 406 of those were reported for the group of vaccinated volunteers and 226 in the group of placebo recipients. Most adverse events were caused by acute respiratory viral infections (>95% in each group), including COVID-19. The study was conducted during the peak period of acute respiratory viral infections. The volunteers were not isolated from the surrounding people, and the possibility of becoming infected could not be excluded. The total number of adverse events related to neither ARVIs nor COVID-19 was 15 in the group of vaccinated volunteers and 6 in the group of placebo recipients. According to the World Health Organization (WHO) criteria, three cases of adverse events were classified as probably related to vaccine administration (adverse events temporally associated with vaccine administration and unlikely to be related to the disease or other medications): acute urticaria on day two after the first vaccination, acute hypotension on day 2, and Quincke’s edema within two hours after the second vaccination. Quincke’s edema was treated by prednisolone injection.

Eighteen cases of adverse events and serious adverse events (12 cases in Group 1 and 6 cases in Group 2) were related to surgical interventions, fractures, or exacerbation of chronic and other diseases and were classified according to the World Health Organization (WHO) criteria as dubiously vaccine-related (adverse events having no temporal relationship with vaccine administration; causality is unlikely but can be explained by an underlying disease or concurrent use of other medications or chemical substances).

The results of the clinical study (phase III) indicate that the EpiVacCorona vaccine is safe. Its administration is predominantly accompanied by mild local reactions.

### 3.3. Immunologic Effectiveness of the EpiVacCorona Vaccine

**Figure 3:**
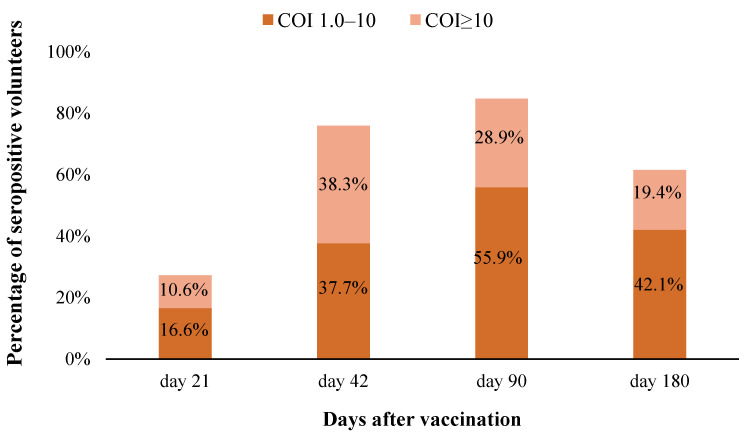
The percentage of seropositive vaccinated volunteers (COI > 1) at different time periods after vaccination.

After the first dose of the EpiVacCorona vaccine had been administered, specific antibodies against antigen components of the vaccine were detected in 27.2% of volunteers on study day 21; the percentage of volunteers having antibodies increased to 76.0% by day 42 and became as high as 84.8% by month three of follow-up. The percentage of seropositive volunteers subsequently decreases, and only 61.5% of volunteers have antibodies by month six of follow-up (Figure 3). 

Three weeks after the first vaccination, the percentage of volunteers with 1.0 ≤ COI ≤ 10 was 16.6%, and the percentage of volunteers with COI ≥ 10 was 10.6%. The largest number of serum samples with COI ≥ 10 (38.3%) was detected on day 42 after the first vaccination; during this period, 37.7% of volunteers had 1.0 ≤ COI ≤ 10. Three months later, the percentage of seropositive volunteers with COI ≥ 10 decreased to 28.9%, and the percentage of those with 1.0 ≤ COI ≤ 10 increased to 55.9%. By the end of the follow-up after 6 months, the percentage of volunteers with 1.0 ≤ COI ≤ 10 decreased to 42.1%, and the percentage of those with COI ≥ 10 was 19.4%.

The vaccination of volunteers with two doses of EpiVacCorona induces the production of SARS-CoV-2 virus-neutralizing antibodies. On day 42 after the first vaccination, virus-neutralizing antibodies (titer > 1:20) were detected in 75.6% of the studied serum samples; the geometric mean titer (GMT) was 1:46 (CI95 = 1:37–1:56). Three months after the vaccination series had been started, virus-neutralizing antibodies were detected in 88.6% of volunteers who did not become infected; the geometric mean titer was 1:74 (CI95 = 1:57–1:95), which is statistically higher than the GMT reached 42 days after the initiation of the vaccination series.

In the group of placebo recipients who had no laboratory-confirmed signs of the coronavirus infection, seroconversion response was not detected in the neutralization assay in all the volunteers. Seroconversion in the neutralization assay was studied for serum samples collected from 100 volunteers, including 50 volunteers vaccinated with EpiVacCorona and 50 placebo recipients at the Clinical Center in Tyumen State Medical University, Ministry of Health of the Russian Federation, Tyumen, Russia. The studies were performed in the research laboratory of the State Research Center of Virology and Biotechnology “VECTOR” of the Federal Service for the Oversight of Consumer Protection and Welfare (Rospotrebnadzor).

### 3.4. Prophylactic Efficacy of the EpiVacCorona Vaccine

The “Multicenter double-blind, placebo-controlled, comparative, randomized trial to assess the tolerability, safety, immunogenicity and prophylactic efficacy of the EpiVacCorona COVID-19 vaccine based on peptide antigens in 3000 volunteers aged 18 years and older (phase III)” was conducted during the period characterized by a rising incidence of COVID-19; the volunteers were not isolated from the surrounding people, and the possibility of becoming infected could not be excluded.

An analysis of paired serum samples In volunteers enrolled in earlier clinical trials (phase I–II) indicated that antibodies appeared in the blood of vaccinated persons 42 days after the first vaccination [21]. Therefore, the prophylactic efficacy was calculated using the data on COVID-19 incidence after the vaccination series had been completed and the antiviral immunity had developed (day 21 after the second vaccination or day 42 after the first vaccination). COVID-19 cases confirmed by laboratory analysis for detecting SARS-CoV-2 markers were included in the calculations.

The laboratory-confirmed SARS-CoV-2 cases were detected in both volunteer groups during the entire follow-up period (Table 2). Volunteers for whom there were protocol violations during the clinical trial (such as non-compliance with the inclusion criteria, meeting the exclusion or withdrawal criteria), as well as volunteers who had been vaccinated with other vaccines or decided to withdraw from the clinical trials, were excluded from calculations. A total of 202 volunteers were excluded from preventive efficacy calculations, including 97 placebo recipients and 105 volunteers who had received the vaccine. Laboratory-confirmed cases of coronavirus infection documented starting day 42 after the initiation of the vaccination series were taken into account for assessing the preventive efficacy of the vaccine.

The preventive efficacy was calculated using the formula:

Preventive efficacy (%) = (1 − OR) × 100

Employing the MedCalc Software Ltd. Odds ratio calculator (Version 20.015) available online [33], where OR = (a/b)/(c/d).

The primary outcomes were the efficacy of the vaccine against symptomatic laboratory-confirmed COVID-19. The total number of volunteers in Groups 1 and 2 with laboratory-confirmed diagnoses of COVID-19 was 259. During the period between day 42 and day 180 after the first vaccination, a total of 147 volunteers were diagnosed with COVID-19, including 58 volunteers who had been vaccinated and 89 placebo recipients. Without taking into account the volunteers excluded from preventive efficacy calculation because of protocol violation, the number of volunteers who had not had COVID-19 starting day 42 after the first vaccination in Groups 1 and 2 was 2090 and 560, respectively. The symptomatic disease preventive efficacy of the EpiVacCorona vaccine for COVID-19 prevention after completion of the vaccination series was 82.5% (CI95 = 75.3–87.6%) (Table 2).

The preventive efficacy of the vaccine during the entire study (day 1 till day 180) was 74.9% (CI95 = 67.4–80.6%). The high vaccine efficacy, including the period up to day 42 (before the vaccination series had been completed), can be attributed to the fact that 27.2% of volunteers in Group 1 had developed a specific humoral response by study day 21 (Figure 3); therefore, the preventive efficacy of the vaccine started to be detected even before vaccination completion.

**Figure 4:**
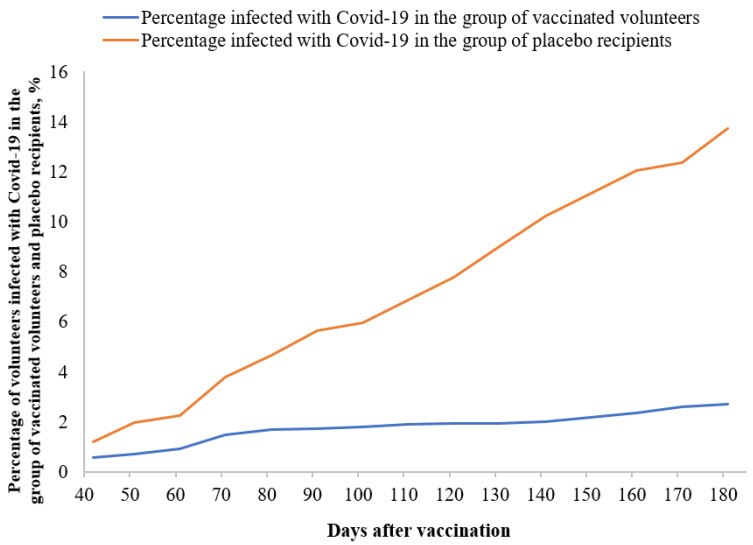
The trends of increasing percentage of volunteers infected with COVID-19 in the group of vaccinated volunteers and placebo recipients.

Figure 4 shows the pattern of changes in the COVID-19 incidence rate after the completion of the vaccination series. The trend of increasing percentage of infected individuals demonstrates that the vaccine remained efficacious during the entire follow-up period, up to day 180.

**Figure 5:**
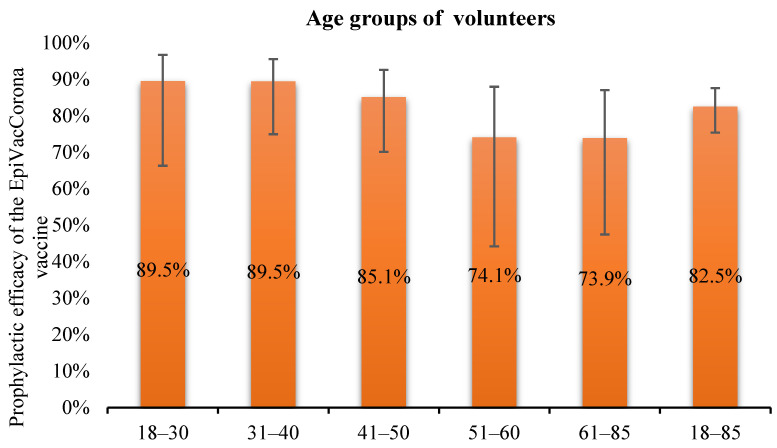
Preventive efficacy of the EpiVacCorona vaccine in different age groups.

The highest efficacy of the EpiVacCorona vaccine (up to 89.5%) was observed in the group of volunteers aged 18–40 years. The preventive efficacy of the vaccine in different age groups is shown in Figure 5.

Vaccine efficacy tended to decrease with increasing volunteers’ age and reached its minimum (73.9%) in the senior age group (>61 years). Preventive efficacy in the age group of 51–60 years was comparable to that in the senior age group (74.1%). This effect was presumably related to the fact that the immune system generally weakens with age. In the age group of 41–50 years, prophylactic efficacy was comparable to the mean prophylactic efficacy determined in this study (85.1%).

The prophylactic efficacy of different age groups was calculated in the same way as it is presented in Table 2.

## 4. Discussion

A “Multicenter double-blind, placebo-controlled, comparative, randomized trial to assess the tolerability, safety, immunogenicity and preventive efficacy of the EpiVacCorona COVID-19 vaccine based on peptide antigens in 3000 volunteers aged 18 years and older” has been conducted.

The objective of this study was to evaluate the tolerability, safety, immunogenicity, and preventive efficacy of the EpiVacCorona vaccine administered in two doses at 21-day intervals.

The post-vaccination process after administration of the study vaccine (EpiVacCorona) and placebo was accompanied by the development of local vaccination reactions (soreness at the injection site, hyperemia, and swelling). The local reactions lasted 2–7 days. No significant intergroup differences in local reactions were observed for vaccinated volunteers and placebo recipients. The severity of systemic reactions was assessed with allowance for acceptable reactions (elevated body temperature, fatigue, and headache). No significant intergroup differences in analyzed parameters were revealed throughout the entire study. The follow-up demonstrated that there were no changes in the functional status of the cardiovascular (arrhythmia, cardiodynia, or tachycardia), respiratory, gastrointestinal (nausea, stomach ache, or vomiting), and urogenital systems (impaired diuresis).

The low frequency and good tolerability of local and systemic reactions demonstrate that the EpiVacCorona is characterized by high safety and weak reactogenicity.

Injection of vaccine products is accompanied by characteristic functional homeostatic shift. Our findings indicate that most parameters remain unchanged after vaccination with EpiVacCorona. Only some hematological and biochemical parameters differed statistically for vaccinated volunteers and placebo recipients but remained within the physiological range. Subsequently, after 3 and 6 months, these parameters did not differ from those in the placebo group. An analysis of findings infers that the EpiVacCorona vaccine has no adverse effects on the functional status of various organs (the liver, the kidneys, the heart, and the pancreas) and systems.

The EpiVacCorona vaccine, administered in two doses within 21–28 days, contributed to developing protective immunity against the SARS-CoV-2 coronavirus. The vaccine induced antigen-specific and neutralizing antibodies. The percentage of seropositive vaccinated volunteers reached its maximum (84.8%) 90 days after receiving the first dose of the vaccine; by day 42, this percentage was 76.0%. The percentage of volunteers with the titer of virus-neutralizing antibodies ≥1:20 by day 42 and day 90 after the first vaccination was 75.6% and 88.6%, respectively.

The preventive efficacy of the EpiVacCorona COVID-19 vaccine in volunteers who had completed the vaccination series was 82.5% (CI95 = 75.3–87.6%). A trend toward decreasing vaccine efficacy in older age groups was presumably related to the fact that systemic immunity weakens with age.

The peptide vaccine EpiVacCorona contains conserved peptide sequences of the spike protein of the SARS-CoV-2 coronavirus, which are not affected by mutations characteristic of new variants of the virus. In the EpiVacCorona vaccine, chemically synthesized antigenic peptides carrying B-cell epitopes are covalently linked to a carrier protein, which is a recombinant chimeric protein containing the nucleoprotein (NP) of the SARS-CoV-2 virus. These conjugate complexes of peptides and carrier proteins are adjuvanted with aluminum hydroxide. Vaccine-induced immunological memory cells are able to recognize conserved epitopes on antigenically altered variants of the coronavirus. T-cell epitopes of NP are conservative for the delta and omicron coronavirus variants and can participate involved in the induction of the immune response of T-lymphocytes specific to various variants of SARS-CoV-2. We designed the EpiVacCorona as a universal vaccine effective against antigenically changing variants of the SARS-CoV-2 virus. The vaccine has a shelf life of one year at a storage temperature of 2–8 degrees. The clinical data obtained indicate good tolerability and low reactogenicity of the EpiVacCorona vaccine. Among volunteers aged 18–60 years, vaccine effectiveness is 82.5% in protecting from symptomatic COVID-19. This significant protection may be explained not only by neutralizing antibodies, which EpiVacCorona induces at low titers but by non-neutralizing antibodies, which play a crucial role in the protection against SARS-CoV-2 via antibody-dependent cellular cytotoxicity (ADCC) and other antibody-dependent mechanisms. Fc-effector functions were associated with mice protection from infection by emerging SARS-CoV-2 variants of concern [34]. Our unpublished data in serum antibodies of non-human primates vaccinated with EpiVacCorona indicated significant ADCC against target VeroE6 cells infected with the SARS-CoV-2 virus. Real-time ADCC measurement was carried out using an xCelligence DP cell analyzer (Agilent Technologies, Santa Clara, CA, USA).

It is interesting to compare the efficacy of this novel EpiVacCorona vaccine against those in clinical use and manufactured in four technological platforms, such as subunit (protein-based) vaccines, mRNA vaccines, adenovirus vector vaccines, and inactivated virus vaccines.

Novavax (NVX-CoV2373) represents a platform of subunit protein vaccine based on the S protein of the SARS-CoV-2. The vaccine is an immunogenic virus-like nanoparticle of recombinant S-protein expressed in Sf9 insect cells [35,36]. NVX-CoV2373 is stored at 2–8 °C. The effectiveness of this vaccine against infection with the original version of SARS-CoV-2 reached 95.6% [35]. In another study of adults who received two doses of this vaccine, the percentage of protection was 89.7% [37]. CoronaVac (Sinovac) is a β-propiolactone inactivated vaccine against COVID-19 [38], developed on Vero cells, adjuvanted with aluminum hydroxide [39]. The vaccine is given in two doses (0.5 mL) 14 days apart [38,39]. CoronaVac is 83.5% effective compared to a placebo in protecting recipients from symptomatic COVID-19 14 days after the second dose of the vaccine among recipients aged 18–59 years [40]. The Sinopharm vaccine (BBIBP) is an inactivated vaccine candidate against the SARS-CoV-2 virus [41]. This vaccine is propagated in Vero cells with subsequent inactivation with β-propionolactone. The resulting inactivated viruses are then combined with aluminum hydroxide adjuvant to increase immunogenicity [42]. The vaccine is given in two doses with 3 weeks interval. It can be stored at normal temperature in a refrigerator [41]. The efficacy of the BBIBP vaccine reached 78.1% against symptomatic SARS-CoV-2 infection 112 days after both doses. The Janssen vaccine (the Johnson and Johnson vaccine) is an adenovirus vector vaccine based on the human adenovirus type 26 [43]. Janssen vaccine is administered as a single dose and can be stored for approximately 2 years at −20 °C or up to 3 months at 2–8 °C [44]. Janssen’s COVID-19 vaccine has been shown to be 66.3% effective in clinical trials in participants not infected with COVID-19. Maximum protection was observed 2 weeks after vaccination [45]. The Oxford-AstraZeneca vaccine is produced using a non-replicating recombinant vector based on a chimpanzee adenovirus (ChAdOx) [46]. The vaccine is stored at a temperature of 2–8 °C. This vaccine is administered in two doses 12 weeks apart or more. The effectiveness of the vaccine reaches 82.4% after the second dose [47]. The ZyCoV-D vaccine contains a circular plasmid DNA that encodes the S protein of the SARS-CoV-2 virus [48]. The vaccine is administered in three doses via a needleless injector [49]. The vaccine is stored at 2–8 °C. The effectiveness of the ZyCoV-D vaccine in clinical trials was 67% [48]. The Moderna vaccine (mRNA-1273) is a liquid nanoparticle mRNA vaccine encoding the S protein. The Moderna vaccine is administered in two doses 28 days apart. A booster dose can also be given 6 months after the second dose [50]. Moderna vaccine should be stored in cold conditions at −25 to −15 °C. After thawing, the vaccine should be kept at 2 to 8 °C for 30 min to 2 h [51,52]. Vaccine efficacy in the age range of 18–65 is 95.6% [50]. Moderna vaccine causes unpleasant side effects such as pain and swelling of the arm at the injection site, fatigue, headache, muscle pain, chills, fever, and nausea [53]. The Pfizer-BioNTech vaccine (BNT162b2) is an mRNA vaccine encapsulated in lipid nanoparticles. The Pfizer vaccine encodes SARS-CoV-2 glycoprotein S [54]. Vaccines are stored at a low temperature of −70 °C, and providing such conditions is a challenge [55]. The vaccine is given in two doses 21 days apart, up to 42 days allowed [56]. The effectiveness of the Pfizer vaccine against COVID-19, measured 28 days after the first dose, reaches 95%. CureVac (CVnCoV) is another mRNA-based vaccine. This vaccine is similar to the Pfizer-BioNTech and Moderna mRNA vaccines and encodes the coronavirus S-protein [57]. Depending on the ambient temperature, the stability of this vaccine is more flexible, with a shelf life of at least 3 months at +5 °C [58]. The vaccine is given in two doses 29 days apart. The efficacy of this mRNA vaccine against symptomatic COVID-19 infection is 47%, which is much lower than the other two mRNA vaccines [59].

Thus, the vaccines currently on the market show protective efficacy against COVID-19 in the range from 47% to 95.6%. Storage conditions for the vaccines also vary significantly from −70 °C to 8 °C. Vaccination with some vaccines causes unpleasant side effects. The analysis of 15 important vaccines, the majority of them we just mentioned above, on criteria such as the dose number, dosing schedule, storage advantages, efficacy, and side effect using the fuzzy MCDM technique, fuzzy PROMETHEE approach, which is an important Multi-Criteria Decision Making (MCDM) technique, concludes that the EpiVacCorona vaccine is the best COVID-19 vaccine due to its efficacy and very low side effect [60]. The use of the proposed techniques will help decisionmaker in the selection process and administration of safe vaccines for the population at large scale.

Therefore, the clinical trials of the EpiVacCorona vaccine for COVID-19 prevention based on peptide antigens indicate that this vaccine has no side effects on the functional status of different organs and systems of the body and is characterized by weak reactogenicity, high immunogenicity, and prophylactic efficacy. The high safety and efficacy of the EpiVacCorona vaccine give grounds for recommending it to be used for regular seasonal prophylaxis of COVID-19 as the safe and efficient vaccine product.

## Figures and Tables

**Table 1:** The percentage of documented cases of local and systemic reactions in volunteers after 3 and 7 days after the first and second vaccination.

Visits	V2 Day 3 after the First Vaccine Dose		V3 Day 7 after the First Vaccine Dose		V6 Day 3 after the Second Vaccine Dose		V7 Day 7 after the Second Vaccine Dose	
Body’s Responses	Vaccine *n* = 2235	Placebo *n* = 740	Vaccine *n* = 2232	Placebo *n* = 739	Vaccine *n* = 2183	Placebo *n* = 688	Vaccine *n* = 2178	Placebo *n* = 684
Soreness at the injection site	500 (22.4%)	183 (24.7%)	31 (1.4%)	10 (1.4%)	271 (12.4%)	89 (12.9%)	31 (1.4%)	9 (1.3%)
Hyperemia	58 (2.6%)	16 (2.2%)	8 (0.4%)	3 (0.4%)	22 (1.0%)	9 (1.3%)	5 (0.2%)	0 (0.0%)
Swelling	50 (2.2%)	17 (2.3%)	5 (0.2%)	5 (0.7%)	26 (1.2%)	9 (1.3%)	4 (0.2%)	1 (0.1%)
Elevated body temperature	57 (2.6%)	21 (2.8%)	32 (1.4%)	11 (1.5%)	33 (1.5%)	15 (2.2%)	30 (1.4%)	10 (1.5%)
Headache	122 (5.5%)	46 (6.2%)	57 (2.6%)	15 (2.0%)	60 (2.7%)	12 (1.7%)	37 (1.7%)	11 (1.6%)
Fatigue	133 (6.0%)	46 (6.2%)	44 (2.0%)	13 (1.8%)	71 (3.3%)	20 (2.9%)	79 (3.6%)	34 (5.0%)

**Table 2:** The incidence of SARS-CoV-2 among volunteers and symptomatic diseases preventive efficacy of the EpiVacCorona vaccine for COVID-19 prevention.

Groups	EpiVacCorona Vaccine	Placebo
The number of volunteers included in assessment of the prophylactic effectiveness of the vaccine	2148	649
The number of volunteers not having COVID-19 on days 42 through 180	2090 ^b^	560 ^d^
The number of volunteers having COVID-19 on days 42 through 180	58 ^a^	89 ^c^
**Calculated prophylactic effectiveness**		
OR	0.175	
Symptomatic diseases preventive efficacy, %	82.5	
CI 95+, %	87.5	
CI 95−, %	75.3	
Z-score	9.954	
Significance level	*p* < 0.0001	

^a^ is the number of vaccinated volunteers having a confirmed diagnosis of COVID-19; ^b^ is the number of vaccinated volunteers who did not have COVID-19; ^c^ is the number of placebo recipients having a confirmed diagnosis of COVID-19; and ^d^ is the number of placebo recipients who did not have COVID-19.

## Data Availability

The datasets generated during and/or analysed during the current study are available from the corresponding author on reasonable request.
